# Caffey's Disease Sans Mandibular and Clavicular Involvement: A Rare Case Report

**DOI:** 10.7759/cureus.1170

**Published:** 2017-04-16

**Authors:** Sachin Khanduri, Gaurav Katyal, Aakshit Goyal, Shreshtha Jain, Tushar Sabharwal, Mriganki Chaudhary

**Affiliations:** 1 Radiodiagnosis, Era's Lucknow Medical College and Hospital

**Keywords:** caffey’s disease, infantile cortical hyperostosis, mandibular, clavicular, unusual

## Abstract

Caffey’s disease, also known as Infantile Cortical Hyperostosis, is a rare, self-limited, benign, inflammatory gene-related disorder of infants that causes bone changes, soft tissue swelling, and irritability. The mandible (75%), clavicles, and ulnae are the bones most frequently involved, others being long bones, lateral ribs, ilia with skull being the rarest. However, we report a case of a 5-month-old male diagnosed with Infantile cortical hyperostosis but with absent mandibular and clavicular involvement, thus depicting the unusual presentation of this disease.

## Introduction

Caffey’s disease is a rare genetic disorder that classically presents with a clinical triad of fever, irritability, and soft tissue swelling in an infant with a radiographical picture of cortical thickening or bony expansion, bridging of bones across interosseous membrane, marginal sclerosis, and hyperostosis of flat bones [1]. The mandible (75%), clavicles, and ulnae are the bones most frequently involved, others being long bones, lateral ribs and iliac bones, the skull being rarest [2]. Diaphyseal involvement and metaphyseal sparing of long bones is noted giving a spindle-shaped appearance of bones.

The clinical importance stems from the fact that certain pathological conditions such as osteomyelitis, bone tumours, scurvy, hypervitaminosis A, and battered baby syndrome can resemble the radiographic findings of Caffey’s disease, thus warranting exclusion.

The purpose of this case report is to emphasize that high index of suspicion must be maintained to diagnose this disease even in cases of an unusual radiological picture.

## Case presentation

The parents of a 5-month-old male patient presented him in the paediatric department of our institution with a history of mild grade fever, generalised body ache, bilateral upper and lower limb swelling, irritability and excessive crying since two weeks, and two episodes of vomiting since one day. There was no associated history of trauma, bleeding from any site or skin rash. The baby weighed about 3.2 kg at birth, born via term normal vaginal delivery, was fully immunized till date, and was exclusively breastfed. The parents gave a negative history for recent travel and their family history was insignificant.

The baby was alert but irritable during the examination, weighed approximately 7.5 kg, and had a head circumference of 40 cm (both measurements around 50th percentile). He had no pallor and icterus, no organomegaly or lymph node enlargement. He had mild grade fever. Other vital signs were normal. There was mild tenderness and swelling in bilateral upper and lower limbs with no accompanying deformities. The swelling was firm to touch without hyperemia or localized raised temperature. The swelling became more prominent over the course of the next three days, though the patient remained afebrile.

He was taken for blood investigations which showed haemoglobin to be 13.5 gm%, total leukocyte count (TLC) 12000/cumm, platelet count 2.5 lacs, erythrocyte sedimentation rate (ESR) raised to 18 (10-15), C-reactive protein (CRP) raised to 18 mg/dl (<10), alkaline phosphatase 250 U/L. Ultrasonography showed abdomen and parotids to be normal.

**Figure 1:**
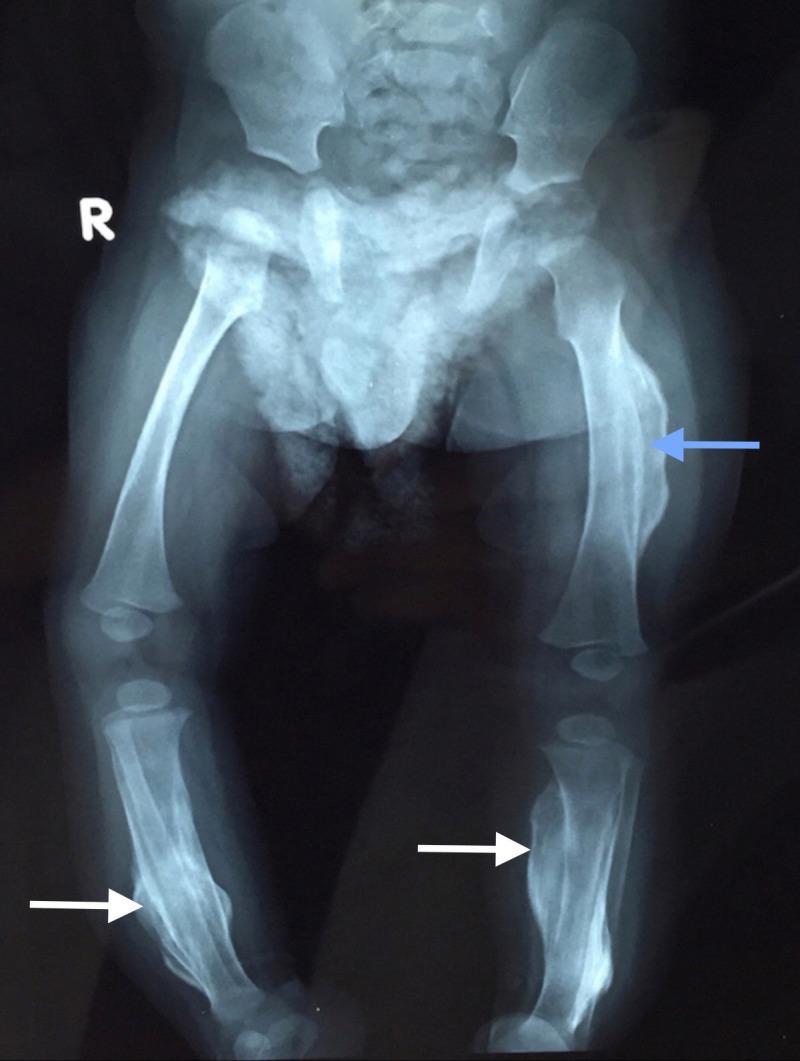
Anteroposterior (AP) view of bilateral lower limbs Radiograph of a 5-month-old male showing complete encasement of left femur by thick lamellated periosteal new bone (blue arrow) confined to diaphysis with epiphyseal and metaphyseal sparing. Similar lamellated two fusiform new bone is also involving diaphysis of bilateral tibia (white arrows).

**Figure 2:**
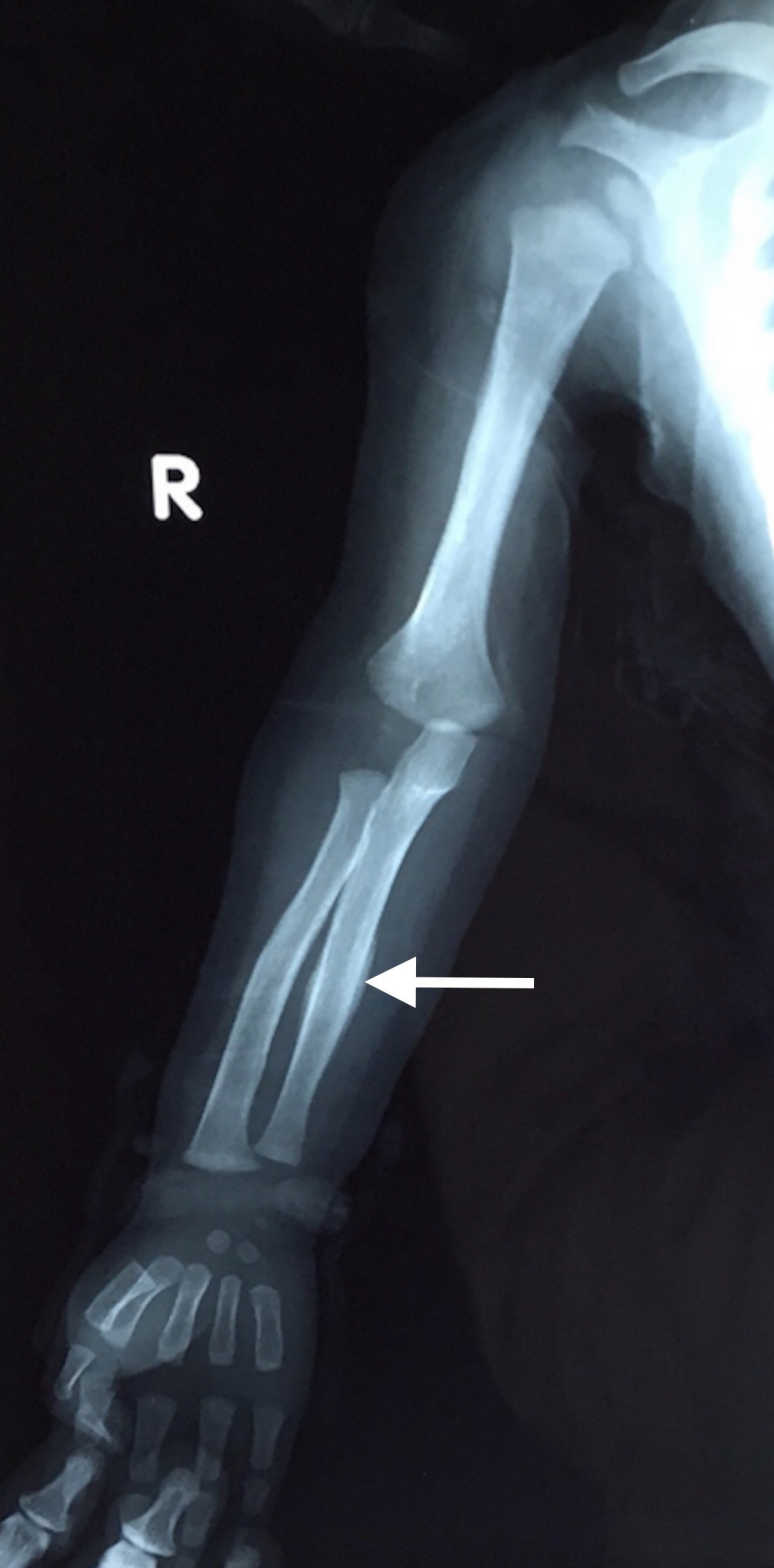
Anteroposterior (AP) view of right upper limb Radiograph shows similar laid down lamellated periosteal reaction involving lateral and medial cortex of ulna (arrow).

**Figure 3:**
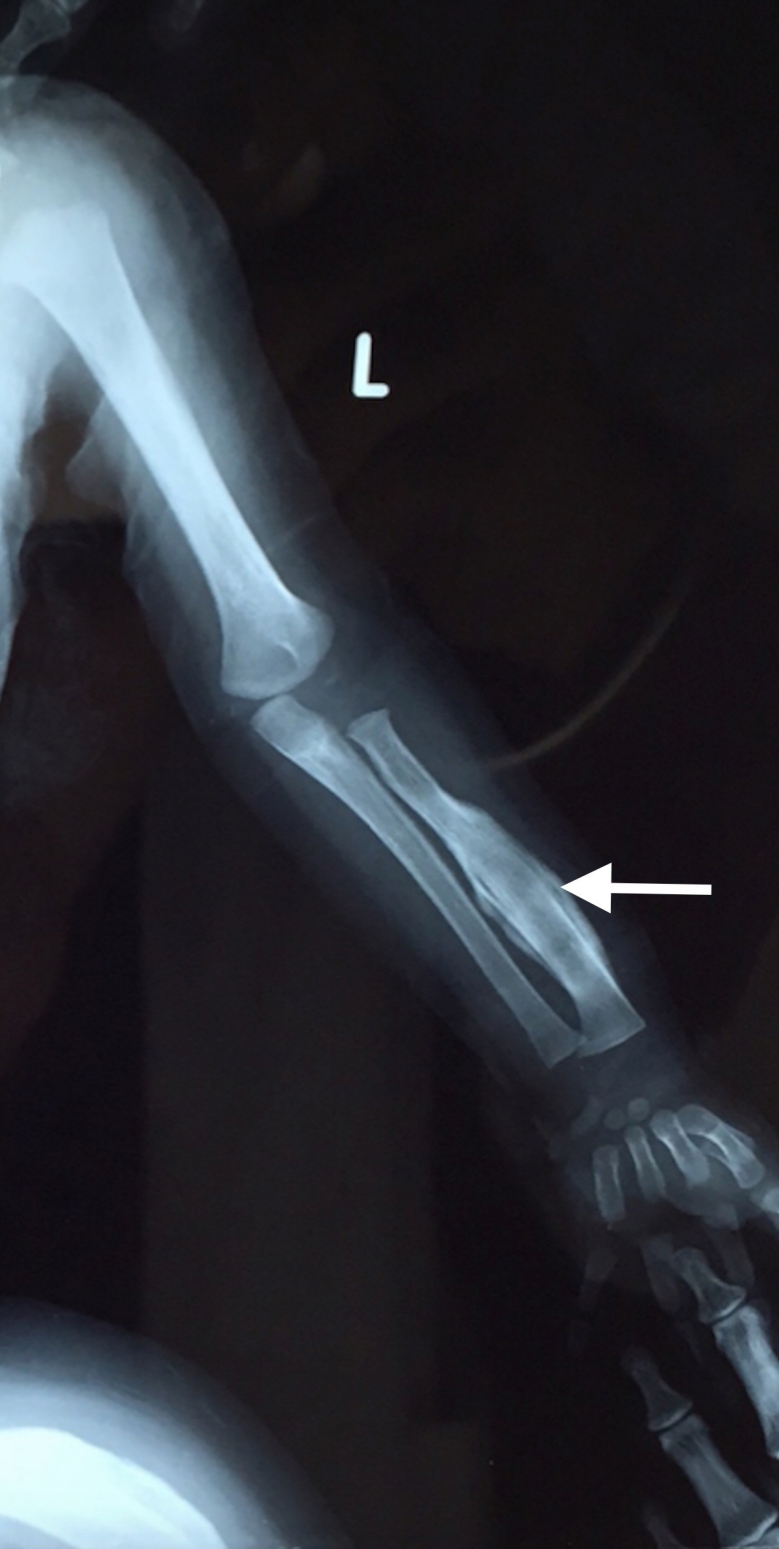
Anteroposterior (AP) view of left upper limb Radiograph shows complete encasement of radius by thick periosteal new bone with resultant cortical widening (arrow). Relative sparing of epiphysis and metaphysis is noted.

**Figure 4:**
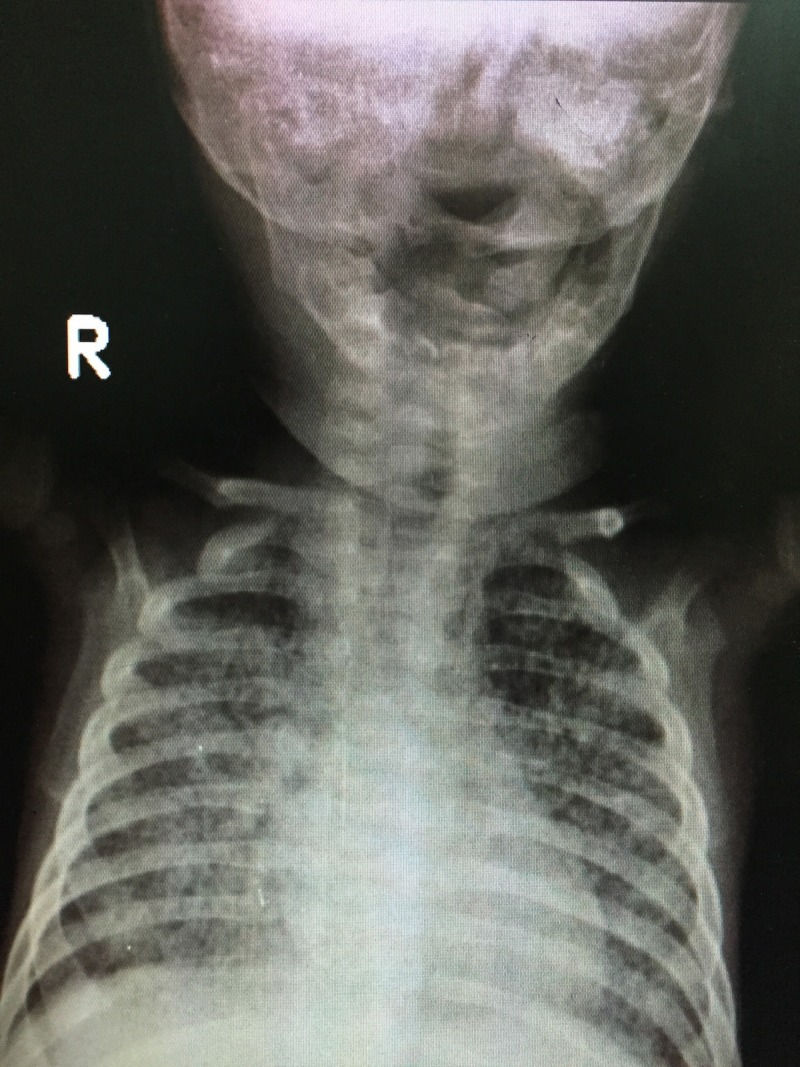
Anteroposterior (AP) view of skull and mandible Radiograph shows no cortical thickening or periosteal new bone formation in the mandible. Bilateral clavicles appear normal.

**Figure 5:**
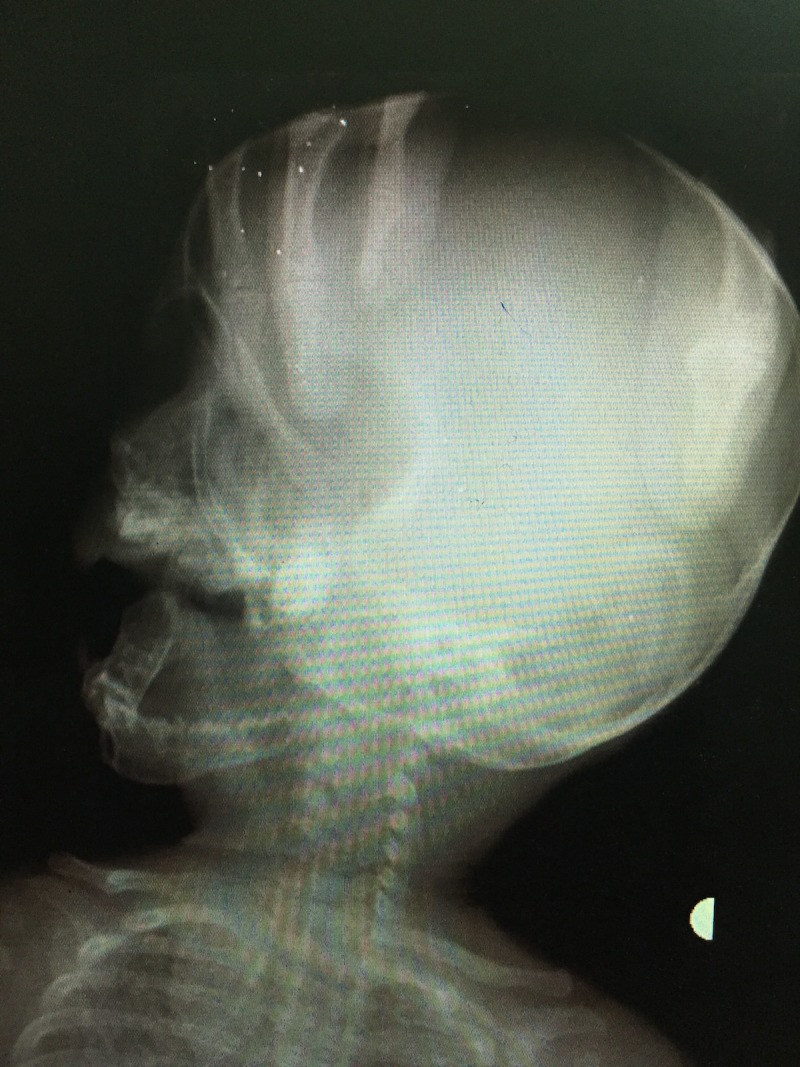
Lateral view of skull and mandible Radiograph shows normal mandible with no cortical thickening. Calvarial bones and clavicle appear normal.

Plain radiographs demonstrated soft tissue swelling with hyperostosis in bilateral tibia, left femur, left radius, and right ulna in an asymmetric fashion. The pattern of bone involvement raised suspicion of Caffey’s disease but absent mandibular and clavicular involvement demanded further evaluation [Figures 1-5].

**Figure 6:**
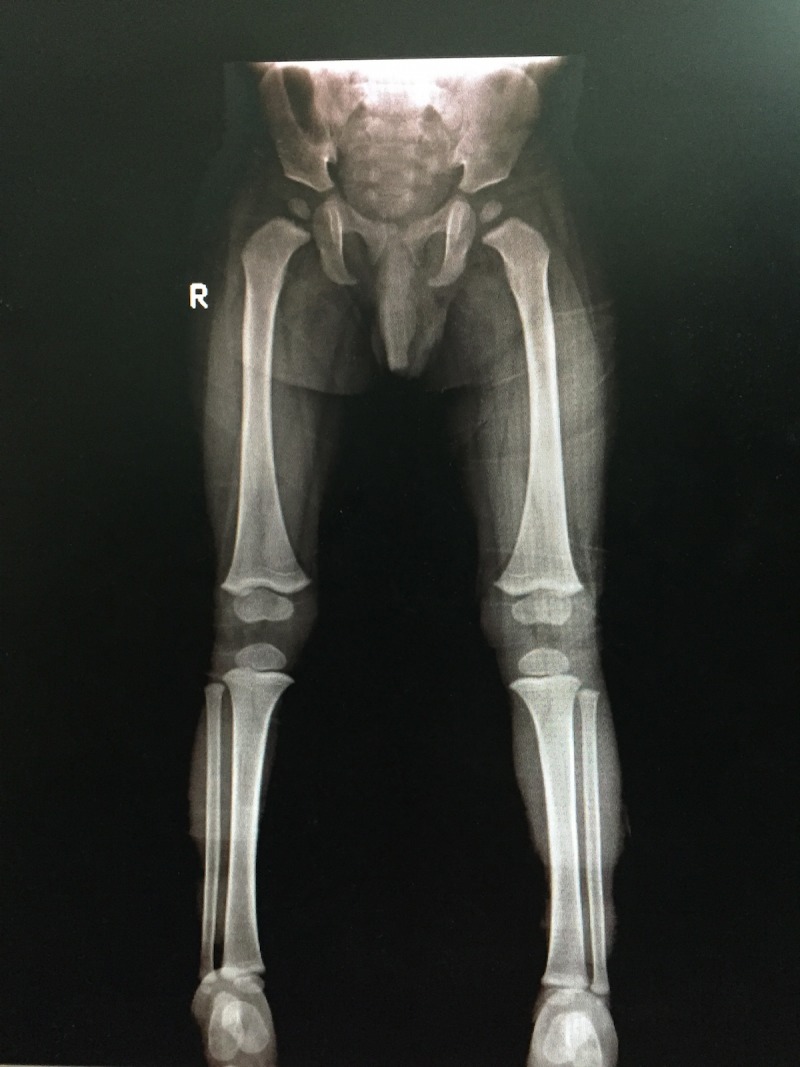
Anteroposterior (AP) view of bilateral lower limbs Follow-up radiograph of the same child after 2.5 years shows improvement with complete resolution of lamellated periosteal bone formation in previously involved left femur and bilateral tibia.

**Figure 7:**
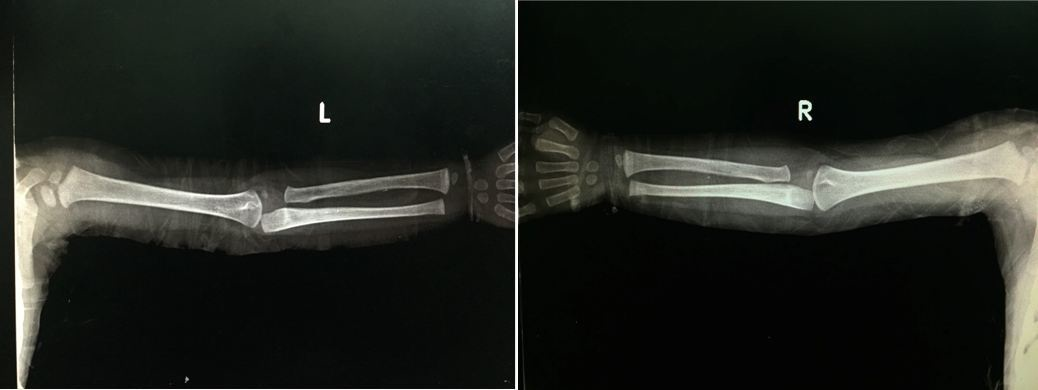
Anteroposterior (AP) view of left and right upper limb Follow-up radiographs of the same child after 2.5 years show complete resolution of periosteal new bone formation in previously involved left radius and right ulna.

The baby was started on Ibuprofen for symptomatic relief of clinical symptoms. Within four weeks the infant showed relief from fever and the bilateral upper and lower limb swelling. Subsequent plain radiographs showed resolving hyperostosis. The child was reviewed again after two years and the X-rays showed a normal picture [Figures 6-7].

## Discussion

Infantile cortical hyperostosis (also known as Caffey’s disease) presents with a clinical picture of hyperirritability, acute inflammation of soft tissues, and profound alterations of the shape and structure of the underlying bones, particularly the long bones, mandible, clavicles, or ribs [2]. It causes bony expansion of the diaphysis presenting itself by five to seven months of age.

The autosomal dominant variant of Caffey’s disease shows a locus on chromosome 17q21. A missense mutation (3040C↠T) in exon 41 of the gene encoding the α1(I) chain of type I collagen (COL1A1) is noted in affected individuals and obligate carriers. It alters the residue 836 (R836C) in the triple-helical domain of this chain. Autosomal recessive form is the more severe and lethal form of this disease [3]. Sporadic cases of infantile cortical hyperostosis have also been explained in addition to the inherited forms. Some of these non-familial cases can be accredited to the administration of prostaglandins E1 and E2 used for the treatment of ductal-dependent cardiac lesions [4].

In theory, two forms of the disease have been recognised: prenatal and infantile. The prenatal form is autosomal recessive, more severe, and rare form of the disease with a poor prognosis. Patients present with angular deformity of the long bones with generalised symmetrical involvement of the skeleton and polyhydramnios [5-6]. The infantile (classical) form is characterized by irritability, soft tissue swelling at various sites (mandible, clavicle, limbs) with local warmth and pain on palpation. Radiographs demonstrate cortical thickening of the affected bony structures [7]. Diaphyseal involvement and metaphyseal sparing of long bones is noted giving a spindle-shaped appearance of bones. Mandible is the most common bone involved followed by clavicle and others [1].

Harris and Ramilo presented a case where mandible involvement occurred late in the disease process [8]. However, in our studied patient, the mandible and the clavicle were not involved during the entire course of the disease. To the best of our knowledge, this is the first reported case in literature with a complete absence of mandible and clavicle involvement.

Some of the important differential diagnosis like osteomyelitis, bone tumours, scurvy, hypervitaminosis A, and battered baby syndrome need to be excluded to diagnose a patient of Caffey’s disease. In our patient, the blood reports were normal which ruled out any metabolic abnormality like hypervitaminosis A and scurvy. There was no history of trauma or any visible skin bruises which undermined the possibility of Battered Baby Syndrome. The absence of fever and subperiosteal collection ruled out osteomyelitis. Our patient presented with generalised body ache and irritability with excessive crying since two weeks and the disease was suspected on an X-ray done in the course of the investigation. Unlike our patient, mandible, clavicles, and ulnae are the bones most frequently involved. However, in our case scenario, the pattern of involvement, the sterile cultures, the absence of subperiosteal collection, and the histopathological finding helped us in forming no other diagnosis but infantile cortical hyperostosis. We followed the patient for two years as the bone changes usually resolve by that time and recurrent episodes are uncommon [9]. Kutty, et al. pointed out that radiography combined with clinical picture is sufficient for its diagnosis and no further investigations are needed [1].

Symptomatic cases require administration of anti-inflammatory drugs such as Naproxen, Indomethacin, Ibuprofen. Steroids can also be used in the cases of poor response to anti-inflammatory drugs [1]. However, our patient responded to Ibuprofen.

## Conclusions

The aim of this report is to highlight the unusual presentation of infantile cortical hyperostosis as in our study case where the patient presented with diaphyseal cortical hyperostosis involving long bones of bilateral upper and lower limbs but with absent mandibular and clavicular involvement. Thus, the high index of suspicion needs to be maintained in such atypical case presentation even after definite exclusion of similar appearing pathologies.
